# ERMP1, a novel potential oncogene involved in UPR and oxidative stress defense, is highly expressed in human cancer

**DOI:** 10.18632/oncotarget.11550

**Published:** 2016-08-23

**Authors:** Alberto Grandi, Alice Santi, Susanna Campagnoli, Matteo Parri, Elisa De Camilli, Chaojun Song, Boquan Jin, Aurelien Lacombe, Serenella Castori-Eppenberger, Paolo Sarmientos, Guido Grandi, Giuseppe Viale, Luigi Terracciano, Paola Chiarugi, Piero Pileri, Renata Grifantini

**Affiliations:** ^1^ Externautics SpA, Siena, Italy; ^2^ Department of Experimental and Clinical Biomedical Science, University of Florence, Florence, Italy; ^3^ Department of Pathology, European Institute of Oncology, Milan, Italy; ^4^ Department of Immunology, The Fourth Military Medical University, Xi'an, China; ^5^ Institute of Pathology, University Hospital Basel, Basel, Switzerland; ^6^ Centre for Integrative Biology - CIBIO, University of Trento, Trento, Italy; ^7^ Department of Oncology and Hemato-Oncology, University of Milan, Milan, Italy; ^8^ INGM Istituto Nazionale Genetica Molecolare, Padiglione Romeo ed Enrica Invernizzi, IRCCS Ospedale Maggiore Policlinico, Milan, Italy

**Keywords:** ERMP1, immunohistochemistry, monoclonal antibody, UPR, oxidative stress

## Abstract

Endoplasmic reticulum (ER) stress and unfolded protein response (UPR) are highly activated in cancer and involved in tumorigenesis and resistance to anti-cancer therapy. UPR is becoming a promising target of anti-cancer therapies. Thus, the identification of UPR components that are highly expressed in cancer could offer new therapeutic opportunity.

In this study, we demonstrate that Endoplasmic Reticulum Metallo Protease 1 (ERMP1) is broadly expressed in a high percentage of breast, colo-rectal, lung, and ovary cancers, regardless of their stage and grade. Moreover, we show that loss of ERMP1 expression significantly hampers proliferation, migration and invasiveness of cancer cells. Furthermore, we show that this protein is an important player in the UPR and defense against oxidative stress. ERMP1 expression is strongly affected by reticular stress induced by thapsigargin and other oxidative stresses. ERMP1 silencing during reticular stress impairs the activation of PERK, a key sensor of the UPR activation. Loss of ERMP1 also prevents the expression of GRP78/BiP, a UPR stress marker involved in the activation of the survival pathway. Finally, ERMP1 silencing in cells exposed to hypoxia leads to inhibition of the Nrf2-mediated anti-oxidant response and to reduction of accumulation of HIF-1, the master transcription factor instructing cells to respond to hypoxic stress. Our results suggest that ERMP1 could act as a molecular starter to the survival response induced by extracellular stresses. Moreover, they provide the rationale for the design of ERMP1-targeting drugs that could act by inhibiting the UPR initial adaptive response of cancer cells and impair cell survival.

## INTRODUCTION

The endoplasmic reticulum (ER) is a multifunctional organelle required for lipid biosynthesis, calcium storage, protein folding and processing. Proper functioning of ER can be affected by a variety of physiological and pathological parameters both inside the cell and in its microenvironment, as well as by different pharmacological agents, causing ER stress. Specific triggers of ER stress include hypoxia, hypoglycemia, hyperthermia, acidosis, calcium or redox imbalances, energy perturbation or fluctuations and others [1]. ER stress severely impacts protein folding, causing an accumulation of unfolded, misfolded or otherwise damaged proteins that can irreparably hamper cellular functions and compromise cell survival. Under such stress, ER evolves a group of signal transduction pathways, collectively termed unfolded protein response (UPR) [1, 2] to restore normal cell function by halting protein translation and activating the signaling pathways that lead to increased production of molecular chaperones involved in protein folding. If ER stress persists, this goal may not be achieved and UPR initiates apoptotic pathways to remove the stressed cells [1–4]. The chaperone GRP78/BiP (78 kDa glucose-regulated protein / Immunoglobulin heavy chain binding protein) and CHOP (C/EBP homology protein) are the most prominent UPR components responsible for the struggle between pro-survival and pro-apoptotic modules, respectively. Under ER stress, GRP78/BiP dissociates from the three ER stress sensors, which include pancreatic ER kinase (PKR)-like ER kinase (PERK), activating transcription factor 6 (ATF6) and inositol-requiring enzyme 1 (IRE1), and binds to the unfolded or misfolded proteins. The dissociation of GRP78/BiP from these stress sensors allows the activation of their pro-survival signaling pathways [1–4].

Accumulating evidence has demonstrated that the ER stress and the UPR are highly induced in various tumors and represent important mechanisms for tumorigenesis and for maintaining malignancy [5]. Since UPR pathways remain in a quiescent state in normal cells, UPR is an emerging target of anti-cancer therapies. Thus, the identification of UPR components that are activated or suppressed in malignancy and the exploration of potential cancer therapeutics targeting the UPR are very active research areas [5, 6].

ERMP1 is a zinc-binding protease belonging to the peptidase M28 family [7]. In the ovary, ERMP1 expression is required for the organization of somatic cells and oocytes into discrete follicular structures [8]. *ERMP1* gene maps at chromosome 9p24, a locus recently described as a novel amplicon in human esophageal and breast cancers [9].

In this study, we identified ERMP1 as a novel broadly tumor-associated-antigen, with high frequency in breast, ovary, lung and colon cancers independently from cancer stages and grades. We demonstrate that ERMP1 protein is involved in cell proliferation, migration and invasiveness. Moreover, we show that ERMP1 is involved in the activation of UPR and in the modulation of GRP78/BiP. Finally, we show that it acts in the defense against oxidative stress. Overall, our results suggest that ERMP1 could be exploited as novel molecular target for the design of drugs perturbing UPR.

## RESULTS

### Discovery of ERMP1 over-expression in human cancers

**Figure 1:**
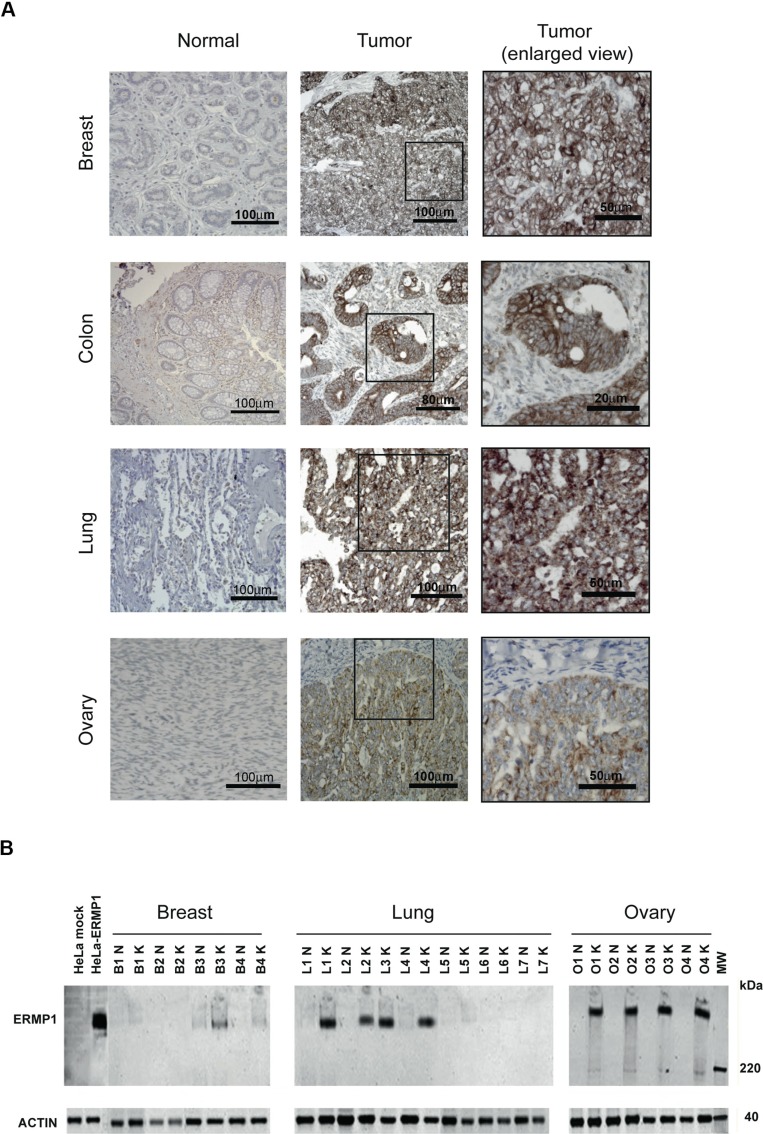
ERMP1 is over-expressed in breast, lung, colon and ovary cancers (**A**) Immunostaining of cancerous and normal samples with the anti-ERMP1 mAb. (**B**) Immunoblot analysis of clinical samples. Total protein extracts (25 μg) from cryo-preserved breast, lung and ovary biopsies of cancer (K) and normal (N) tissues from patients were separated by SDS-PAGE and subjected to Western blot with anti-ERMP1 or anti-actin mAbs. Total extracts from HeLa cells transfected with ERMP1 coding plasmid and mock-transfected (empty plasmid) cells were analyzed in parallel as controls. Molecular weight markers (MW) are on the right.

We have recently described the validation and use of the YOMICS^@^ murine polyclonal antibody library (http://www.yomics.com/), to discover tumor markers by IHC analysis [10, 11]. During the screening of the entire antibody library on tissue microarrays (TMAs) carrying cancerous and normal formalin-fixed paraffin-embedded (FFPE) samples from breast, colon, lung, and ovary samples, we found that the pAb687-YOM, a polyclonal antibody raised against a recombinant ERMP1 domain (amino acid 1–204) (rERMP1) specifically detected the expression of its target protein in cancer samples of the four anatomical sites whereas it gave a negligible staining in the corresponding normal tissues (Supplementary Figure S1), suggesting that ERMP1 is expressed at higher level in breast, colon, lung, and ovary cancers. A mouse monoclonal antibody (ERMP1 mAb) raised against rERMP1 by the conventional hybridoma technology and specific for rERMP1 (full details about the fine specificity are given below) was used to confirm ERMP1 expression in cancer tissues. In a first step a TMA carrying five duplicate tumor and the corresponding normal samples for each tumor type (breast, colon, lung, and ovary) were analyzed for their ERMP1 expression. ERMP1 mAb specifically stained breast (4/5 positive), colon (3/5 positive), ovary (4/5 positive) and lung (3/5 positive) cancers, with a concomitant negligible staining in the corresponding normal samples. Afterwards, IHC analysis was extended to TMA carrying 43 to 47 FFPE samples per each tumor entity. The ERMP1 mAb showed positive staining in breast (94%), colon (94%), lung (74%), and ovary (96%) cancer samples. Most of them showed a moderate or strong intensity (frequencies ranging from 59.6 to 76.6%). In general, the staining was quite homogenous (50–100% of cells were stained by the mAb in 70% of samples) and cytoplasmic, though in some samples it also decorated the plasma membrane (Figure 1A).

The specificity of the ERMP1 mAb was verified by ELISA on rERMP1 (data not shown) and by Western blot on HeLa cells transfected with full-length ERMP1 cDNA. As shown in Supplementary Figure S2, ERMP1 mAb specifically detected a main band at around 300 kDa (higher than expected) on total protein extracts of ERMP1-transfected HeLa cells, previously separated by SDS-PAGE under reducing conditions, which was not visible in HeLa cells transfected with the “empty” pcDNA3. 1D plasmid. The high MW band was also detected by pAb687-YOM (Supplementary Figure S2). Though not investigated, the apparently aberrant ERMP1 MW could be ascribed to the formation of ERMP1 stable multimers or complexes with unknown molecule(s), which are not dissociated under the used conditions. To further assess ERMP1 over-expression in cancer, Western blot was carried out on total protein extracts (25 μg) of cancer and matched normal samples derived from cryopreserved surgical resections of breast (4 patients), lung (7 patients) and ovary (4 patients). As shown in Figure 1B, the high MW band, similar in size to that detected in ERMP1-transfected HeLa cells, was clearly visible in 2/4 breast cancer samples, in 4/7 lung cancer samples and in 4/4 ovary cancer samples, but not or only marginally visible in the corresponding normal samples.

We additionally investigated ERMP1 mRNA level in clinical samples from breast, lung, colon, ovary and prostate samples by q-RT-PCR (2 normal samples and 2-6 tumor samples for each organ). ERMP1 transcript was detected in all tested samples. It was found up-regulated in all colon cancer and prostate cancer samples, and in 50% of lung, ovary and breast cancer (fold increase versus normal samples ranging from approximately 2 to 13) (Supplementary Figure S3).

Finally, we investigated ERMP1 expression in normal tissues by IHC using a TMA carrying normal tissues from 33 anatomical districts (MNO961). In most tissues the ERMP1 mAb staining was negligible or negative (Supplementary Figure S4).

### Prevalence of ERMP1 in breast, lung, ovary and colon cancers

To study the prevalence of ERMP1 and its potential clinical significance IHC analysis with ERMP1 mAb was performed on TMAs carrying different sets of well-characterized FFPE samples from breast (136 cases, in duplicate), colon (CRC) (667 cases), lung (368 cases of Non-Small-Cell Lung Carcinoma (NSCLC) and ovary (150 cases) cancers, selected on the basis of the availability of relevant clinical and molecular data (Supplementary Table S1). In general, the staining was quite homogenous and predominantly cytoplasmic with sporadic accentuation on plasma membrane.

In breast cancer, ERMP1 was detected in 94.2% of the cases, 42.3% showed a strong or moderate staining (see Materials and Methods). An analysis stratified on available clinico-pathological data (Table 1) showed that the frequency of the protein expression was independent from tumor stage, histological grading and hormonal receptors as well as HER2 status (Table 1). Interestingly, ERMP1 showed a high/moderate staining in 57% of the triple negative (HER2-, ER-, PR- negative) breast cancer samples (Table 1).

**Table 1:** Frequency of ERMP1-positive cancer samples

				ERMP1 positive		ERMP1 moderate or high intensity	
CANCER	Parameter	Scoring	N° of samples	N° of samples	%	N° of samples	%
BREAST	pT	1&2	110	106	96.4	49	44.5
		3&4	23	20	87.0	7	30.4
	pN	0&1	107	101	94.4	48	44.9
		2&3	12	12	100.0	3	25.0
	Grade	1&2	74	69	93.2	30	40.5
		3	63	60	95.2	28	44.4
	Her2	+	15	14	93.3	4	26.7
		−	121	113	93.4	54	44.6
	ER	+	97	92	94.8	39	40.2
		−	39	36	92.3	19	48.7
	PR	+	34	31	91.2	15	44.1
		−	89	84	94.4	39	43.8
	Receptor profile	Her2^+^ER^+^PR^+^	1	1		0	
		Her2^+^ER^+^PR^−^	5	5		2	
		Her2^+^ER^−^PR^−^	6	5		1	
		Her2^−^ER^+^PR^+^	33	30	90.9	15	45.5
		Her2^−^ER^+^PR^−^	50	48	96.0	20	40.0
		Her2^−^ER^−^PR^−^	28	26	92.9	16	57.1
COLON	pT	1&2	132	131	99.2	111	84.1
		3&4	513	508	99.0	415	80.9
	pN	0&1	511	503	98.4	420	82.2
		2	137	131	95.6	103	75.2
	Grade	1&2	613	600	97.9	493	80.4
		3	39	38	97.4	33	84.6
	K-RAS	Wt	180	175	97.2	152	84.4
		Mut	84	83	98.8	70	83.3
	B-RAF	Wt	230	225	97.8	193	83.9
		Mut	24	23	95.8	18	75.0
LUNG	pT	1&2	368	351	95.4	153	41.6
	Grade	1&2	198	187	94.4	85	42.9
		3&4	158	153	96.8	63	39.9
	Ki67	0&1	144	137	95.1	72	50.0
		2&3	202	194	96.0	81	40.1
OVARY	pT	1&2	20	18	90.0	6	30.0
		3	123	120	97.6	45	36.6

Staining frequency was not calculated for groups with less than 10 samples.

In CRC, ERMP1 was detected in 97.9% of the cases, 80.7% showed a strong or moderate staining. Also in this cancer type, the protein expression was found to be independent from pT and pN stages (Table 1). In CRC ERMP1 expression was also independent from the mutational state of the V-Ki-ras2 Kirsten rat sarcoma viral oncogene homolog (*KRAS*) and v-Raf murine sarcoma viral oncogene homolog B (*BRAF*) genes. Detailed information regarding KRAS and BRAF analysis of tumor samples are summarized in Supplementary Table S1.

In NSCLC cancer, ERMP1 was detected in 95.4% of the cases, 41.4% of which showed a strong or moderate staining. Also in this cancer type, no association was found between protein expression levels and pathological features including grading and proliferation index as assessed by Ki67 immunostaining (Table 1).

In ovarian cancer, ERMP1 was detected in 96.7% of the samples, with a strong to moderate staining in 35.3% of them and without association with pT stages (Supplementary Table S1).

### ERMP1 is expressed intracellularly in cancer cell lines

**Figure 2:**
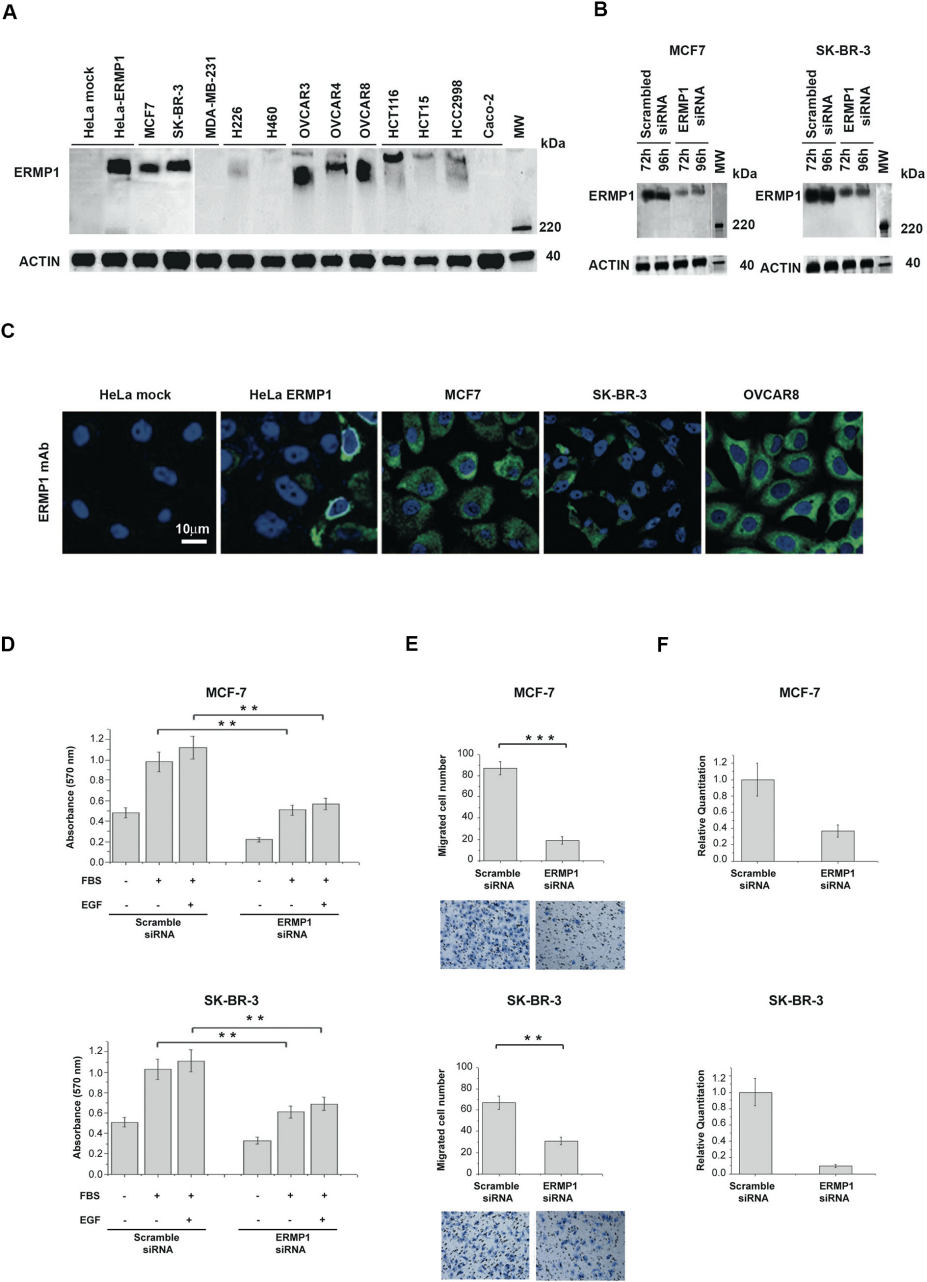
ERMP1 is expressed in human cancer cell lines and is involved in cell proliferation and invasiveness (**A**) ERMP1 expression in cancer cells lines. Total extracts of the indicated cancer cell lines were separated on SDS-PAGE (25 μg/lane, corresponding to approximately 0.5 × 10^6^ cells) and subjected to Western blot using the anti-ERMP1 mAb. Total extracts from HeLa cells transfected with ERMP1 coding plasmid and mock-transfected (empty plasmid) cells were analyzed in parallel as controls. Images derive from distinct immunoblots. (**B**) ERMP1 knockdown by siRNA. MCF7 and SK-BR-3 cells were transfected with 1nM of ERMP1-specific siRNAs or scramble controls. After 72 and 96 hours, total protein extracts were separated by SDS-PAGE, blotted on nitrocellulose membranes and subjected to Western blot using the anti-ERMP1 mAb, and an anti-actin antibody as internal control. (**C**) ERMP1 localization in cancer cells lines. Confocal microscopy analysis of cells fixed, permeabilized and incubated with the anti-ERMP1 mAb. HeLa cells transfected with ERMP1 coding plasmid and mock control cells were analyzed in parallel. Cells were stained with Alexafluor 488-labeled goat anti-mouse antibodies to detect ERMP1 (green) and DAPI to visualize nuclei (blue). (**D**) Cell proliferation. MCF7 and SK-BR-3 cells were transfected with ERMP1-specific or scrambled siRNA. After 48 hours cells (2 × 10^4^ /well) were plated in 96 well plates and incubated for additional 24 hours in medium containing 2.5% FBS, with or without 10ng/ml EGF or in stimuli-free medium. Thereafter proliferation was assessed by the MTT assay. (**E**) Cell invasiveness. MCF7 and SK-BR-3 cells transfected with ERMP1-specific siRNA or scrambled siRNA were loaded of matrigel-coated 96-well plates and analyzed by the Boyden invasion assay. Cells migrated towards the lower surface of the chamber filters were fixed and counted after Diff-Quick staining. Images of the visual counting of each sample are reported below the graphs. Asterisks mark samples showing a significant difference (**p < 0.05; ***p < 0.01). (**F**) Reduction of ERMP1 transcript level in MCF7 and SK-BR-3 cells upon gene silencing. q-RT-PCR was performed using *MAPK* gene as housekeeping control. Data from phenotypic assays represent the means of three replicated experiments, run in triplicate.

IHC data showed that ERMP1 is highly expressed in breast, colon, lung and ovary cancers. Therefore we assessed ERMP1 expression in a panel of cell lines derived from these tumors by Western blot using the ERMP1 mAb. Among the cell lines tested, the high MW ERMP1 band was detected in 9 cell lines including the breast cell lines SK-BR-3 and MCF7, the lung cell line H226, the colon cell lines Colo205, HCT116 and HCC2998, and the ovary cell lines OVCAR3 OVCAR4 and OVCAR8 (Figure 2A) whereas the same band was absent or weak in the other tested cell lines. The specificity of the immune-reactive band was further confirmed by gene silencing using the RNA interference technology. SK-BR-3 and MCF7 cells were transfected with four different ERMP1-specific siRNAs or with a scrambled siRNA and 72 hours later the reduction of ERMP1 mRNA level was assessed by q-RT-PCR. Transfection with 10 nM or 50 nM of ERMP1 siRNAs caused a substantial loss of cell adherence and viability (see paragraph below). When used at 1nM, ERMP1 mRNA was significantly reduced at similar level by the four ERMP1 specific siRNAs (Supplementary Figure S5). Western blot analysis of total cell lysates 72 and 96 hours after transfection of siRNA showed that ERMP1 silencing markedly reduced the ERMP1 immuno-reactive band (Figure 2B). A similar effect was obtained with all tested siRNAs (data not shown).

We then assessed ERMP1 localization by immunostaining and confocal microscopy analysis of selected cancer cell lines previously found to be ERMP1 positive by Western blot. Figure 2C shows that in MCF7, SK-BR-3 and OVCAR8 cells, the ERMP1 protein is localized intracellularly and it partially accumulates in the perinuclear region. Similar localization results were obtained in HeLa cells transfected with ERMP1 cDNA, run in parallel as control. This result is in agreement with its annotation of protein associated to the ER [8].

### ERMP1 depletion reduces cell proliferation and invasiveness of cancer cell lines, but it does not influence apoptosis

We then investigated the role of ERMP1 in processes important for tumor development, such as proliferation, apoptosis and invasiveness, by gene silencing of SK-BR-3 and MCF7 cell lines with ERMP1-siRNAs or scramble siRNAs (1 nM). Proliferation analysis was assessed by the MTT assay on siRNA-treated cells cultured either in serum-free medium (starvation medium), or added with 2.5% FCS, with our without 10 ng/ml EGF as proliferative stimuli and analyzed by the MTT assay. A 2-fold reduction of cell proliferation was observed in all tested culture conditions (Figure 2D) subsequent to ERMP1 silencing.

Cell invasiveness was then assessed *in vitro* by the Boyden assay. ERMP1-silencing induced a marked reduction of the invasive phenotype of both cell lines, compared to control samples (Figure 2E). Moreover, the effect of ERMP1 in apoptosis was assessed by the Annexin V- PI staining. ERMP1 silencing did not alter the percentage of apoptotic cells (data not shown).

Since ERMP1 is involved in the cellular response to hypoxia, as described in the following paragraphs, the effect of ERMP1 silencing on cell proliferation was assessed in MCF7 and SK-BR-3 cells cultured for 24 hours in hypoxic conditions (1% O_2_), as compared to cells maintained in normoxia (20% O_2_). The inhibition of cell proliferation caused by loss of ERMP1 was similar in hypoxia and normoxia (Supplementary Figure S6), indicating that the ERMP1 role in cell proliferation is independent from oxygen availability.

### ERMP1 expression is modulated by endoplasmic reticulum and reactive oxygen species oxidative stresses

**Figure 3:**
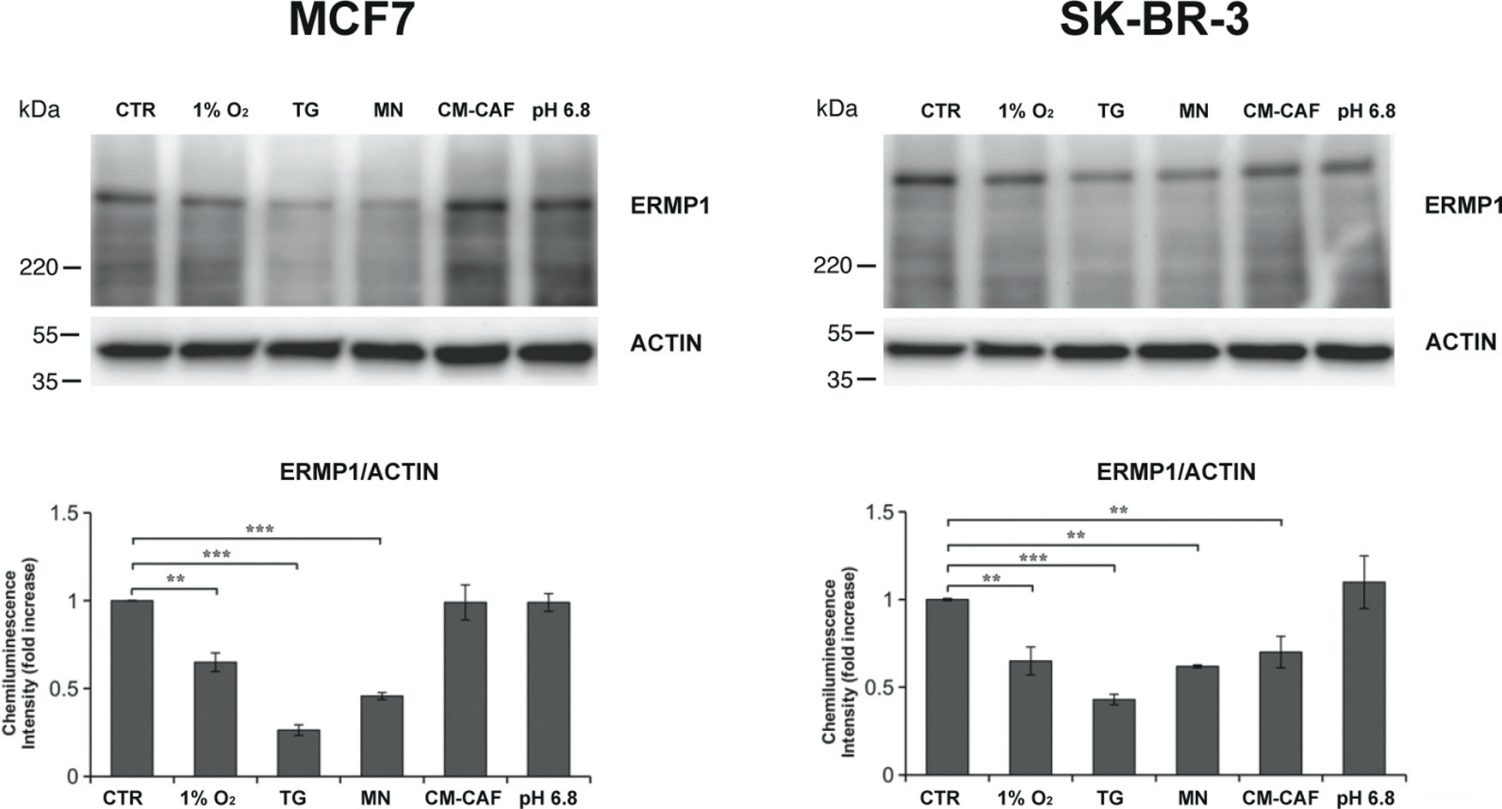
ERMP1 expression is modulated by tumor microenvironment stimuli Western blot analysis of ERMP1 expression in response to oxygen availability, pH and oxidative stresses. MCF7 (left) and SK-BR-3 cells (right) were cultured for 24 h under hypoxia (1% O_2_), 0.5 μM thapsigargin (TG), 3 μM (MN) menadione, acidic medium (pH 6.8), or with conditioned media (CM) from cancer-associated fibroblasts (CAFs). Total cells extracts were separated by SDS-PAGE (25 μg/lane) and analyzed by Western blot with the anti-ERMP1 and actin-antibodies. Graphs below the immunoblots represent a relative quantification of ERMP1, expressed as chemiluminescence intensity ratio of ERMP1 vs actin bands. Data represent the meanvalues of four replicated experiments (**p < 0.05 and ***p < 0.01).

The identification of the regulatory or environmental conditions that promote ERMP1 expression could contribute to elucidate its role in cancer cells. To this purpose, we exposed MCF7 and SK-BR-3 cells to a series of stimuli that induce structural and metabolic cell changes and monitored ERMP1 expression, as compared to cells grown in standard conditions. Cells were either grown for 24 hours under hypoxia (1% O_2_), ER stress (0.5 μM thapsigargin) or reactive oxygen species stress (3 μM menadione) or acidic stress (pH 6.8). In parallel, to mimic the effect of tumor microenvironment, cancer cells were also co-incubated for 24 hours with the conditioned media (CM) from stromal cells known to be involved in cancer progression, such as cancer-associated fibroblasts (CAFs). Western blot analysis of total cell extracts showed in Figure 3 indicates that exposure to hypoxia, thapsigargin and menadione significantly decreased ERMP1 expression in both cell lines, with thapsigargin causing the most marked effect. Cell exposure to CM-CAFs decreased ERMP1 expression only in SK-BR-3 cells. Acidic stress did not alter ERMP1 expression. ERMP1 transcription analysis in MCF7 and SK-BR-3 cells under hypoxia, thapsigargin and menadione by q-RT-PCR did not show any significant alteration of ERMP1 transcript level, as compared to untreated cells (data not shown).

### ERMP1 is involved in the UPR and the response to oxidative stress

**Figure 4:**
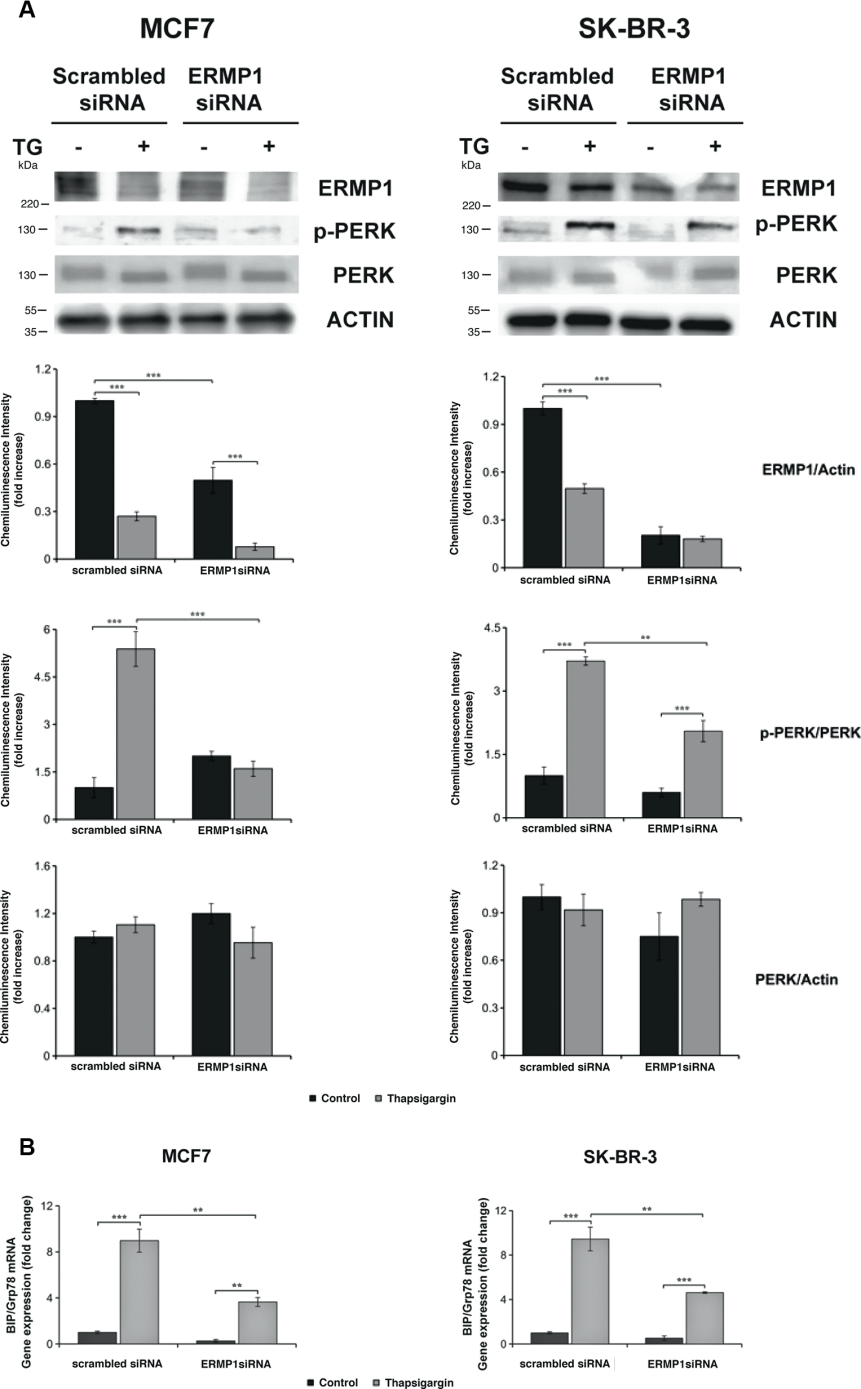
ERMP1 controls the ER stress-induced activation of PERK and GRP78/BiP (**A**) Western blot analysis of PERK phosphorylation in response to ERMP1 silencing and thapsigargin stress. MCF7 and SK-BR-3 cells knocked down for ERMP1 gene or non-silenced (scrambled siRNA) were treated with 0.5 μM thapsigargin (TG) for 24 hours in starvation medium, lysed, and cell lysate were separated by PAGE-SDS and used for Western Blot analysis. The membranes were treated with anti-ERMP1, anti-p-PERK, anti-PERK and anti-actin antibodies. (**B**) q-RT-PCR analysis of GRP78/BiP transcription in MCF7 and SK-BR-3 cells subsequent to ERMP1-silencing and/or exposition to thapsigargin stress. Total RNA was isolated from the MCF7 and SK-BR-3 the same cell samples and GRP78/BiP transcription was analyzed by q-RT-PCR, using β2-microglobulin as reference gene for sample normalization. Data are expressed as means ± S.D. calculated on an average of four independent experiments (**p < 0.05 and ***p < 0.01).

The effect of thapsigargin, menadione and hypoxia on ERMP1 expression in cancer cells led to the hypothesis that ERMP1 is involved in the UPR and in the response to oxidative stress. We addressed ERMP1 role in UPR by ERMP1 silencing in SK-BR-3 and MCF7 cells, and monitoring the activation of PERK, inferred by the accumulation of the phosphorylated form of the protein, and the expression of GRP78/BiP, two fundamental UPR mediators. We found that ERMP1 knock-down inhibits PERK phosphorylation, as judged by Western blot analysis of protein cell extracts using antibodies able to discriminate the PERK phosphorylated and non-phosphorylated forms (Figure 4A). Moreover, ERMP1 knock-down reduces GRP78/BiP transcription level, as judged by q-RT-PCR analysis of total RNA purified from the cell samples (Figure 4B), thus confirming that ERMP1 is involved in the activation of the signaling pathway responsible for ER homeostasis.

**Figure 5:**
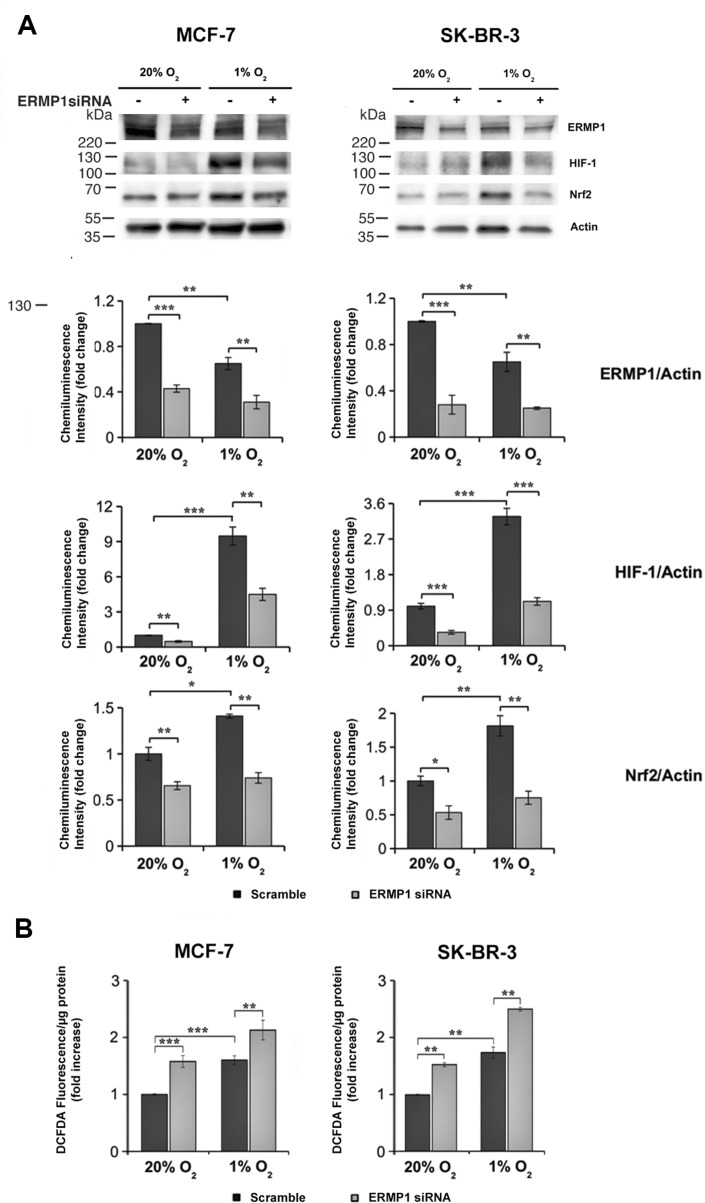
ERMP1 contributes to the cellular response to oxidative stress (**A**) Western blot analysis of ERMP1 HIF-1, and Nrf2 in response to ERMP1 silencing and oxygen availability. MCF7 and SK-BR-3 cells (left and right, respectively) knocked down for ERMP1 gene or non-silenced were cultured for 24 hours at 20% O_2_ and at 1% O_2_, and cell extracts were used for Western Blot analysis with anti-ERMP1, anti-HIF-1, anti-Nrf2 and anti-actin antibodies. Graphs below each immunoblot represent the relative quantification of the ERMP1, HIF-1, and Nrf2 expression vs actin, obtained by averaging chemiluminescence band intensity data, representative of 5 independent experiments. (**B**) ERMP1 silencing causes accumulation of intracellular ROS. The level of intracellular ROS in ERMP1-silenced MCF7 and SK-BR-3 cells cultured at 20% O_2_ and 1% O_2_ was evaluated with DCFDA. Data are expressed as means ± S.D. calculated on an average of four independent experiments (*p < 0.1; **p < 0.05 and ***p < 0.01).

In order to investigate the ERMP1 role in the response to oxidative stress, we assessed the expression of the nuclear factor-E2-related factor-2 (Nrf-2) and the hypoxia inducible factor 1 (HIF-1), two master effectors of oxidative stress response, in SK-BR-3 and MCF7 cells treated with ERMP1 siRNAs and maintained for 24 hours in normoxia (20% O_2_) or hypoxia. We found that ERMP1 knockdown significantly decreases the expression of HIF- 1 protein levels (Figure 5A) under both O_2_ conditions. In contrast, Nrf2 expression was significantly reduced under hypoxia, while it changed only marginally under normoxia. An analysis of the intracellular level of reactive oxygen species (ROS) in these samples using the DCFDA assay showed a marked increase of ROS both at 20% O_2_ and 1% O_2_, compared to their non-silenced counterparts (Figure 5B).

## DISCUSSION

*ERMP1* gene (alias *FxnA* and *KIAA1815)* encodes a metallopeptidase belonging to the M28 family of approximately 100 kDa, with nine predicted transmembrane domains normally localized in the ER. In the rat ERMP1 gene is expressed in granulosa cells and its abundance is maximal 48 hours after birth, i.e. during the initiation of follicular assembly [8]. Rat ERMP1 is required for the organization of somatic cells and oocytes into discrete follicular structures, since loss of ERMP1 causes substantial loss of follicles, and structural disorganization of the ovary, with many abnormal follicles containing more than one oocyte and clusters of somatic cells not associated with any oocytes. ERMP1 role in the follicular organization seems to be ascribed to its proteolytic activity on precursor proteins required for intra-ovarian cell-to-cell communication [8]. Studies in human esophageal and breast cancers showed that ERMP1 gene is located in the 9p24 genomic amplicon. In breast cancer the 9p24 genomic amplicon contains six genes, among which *ERMP1* and *IL33* are overexpressed independently of the copy number increase, while *GASC1, UHRF2, KIAA1432* and *C9orf123* are overexpressed only in the context of gene amplification [9]. Accumulating evidences support the notion that the 9p24 amplicon contains candidate oncogenes. Indeed, *GASC1* and *UHRF2* encodes epigenetic factors involved in tumorigenesis [9, 12–15]. Concerning ERMP1, its role in cancer was so far unknown.

The present study brings a substantial contribution to our current knowledge on ERMP1 and provides robust experimental evidence of its association with cancers with high morbidity and mortality rate. By an extensive IHC investigation we discovered that the protein is highly expressed in a large fraction of breast, colon, lung, and ovary cancers (frequency ranging from approximately 94% to 98%), among which, the highest expression level (as judged by samples with strong/moderate IHC staining intensity) was found in colon cancer (80.7%), compared to lung, breast cancer and ovary cancer (41.4%, 42.3%, 35.3% and respectively). In accordance, ERMP1 transcript was found upregulated in these cancer types. A prevalence analysis in these four tumor types showed that ERMP1 is expressed at similar level in early and late stages as well as in highly and poorly differentiated cancers. Interestingly ERMP1 expression seems to be independent from important predictive biomarkers in breast as well as in colon cancer. In breast cancer, ERMP1 is expressed at similar frequency regardless of the expression of HER2, progesterone and estrogen receptors. Importantly, it is expressed in a significant fraction of triple negative breast cancer samples (93% positivity, 57% of which with moderate or high intensity). In colon cancer, ERMP1 is expressed irrespective of *KRAS and /or BRAF* mutational status. Although not deeply investigated, our q-RT-PCR data indicate that ERMP1 is also upregulated in prostate cancer. Moreover, public transcriptome results available at the Oncomine database (https://www.oncomine.org), score ERMP1 among the top 10% over-expressed genes in breast and liver cancer (data from 5 and 2 studies related to cancer-versus-normal comparisons, respectively).

In agreement with its predicted localization we found ERMP1 in the intracellular compartment of cancer cells by IHC and confocal microscopy. ERMP1 migrates in SDS-PAGE with an unexpected MW of approximately 300 kDa, suggesting that it could form stable homomultimers or complexes with unknown molecule(s), which are not dissociated under the denaturing and reducing conditions used in Western blot.

The evidence that ERMP1 is a broadly and highly expressed in several cancer types suggests that it plays an important role in cell processes and pathways crucial for cancer development. Our *in vitro* studies strongly support this hypothesis and indicate that ERMP1 acts as an important player in ER stress and UPR, as well as in the defense against oxidative stress. Although a full understanding of ERMP1 function in the UPR pathway and in the defense to oxidative stress would deserve more in-depth studies, our working hypothesis is that ERMP1 contributes as a molecular starter to the survival response induced by extracellular stresses. This hypothesis is based on five major observations. First, loss of ERMP1 expression caused by transient ERMP1 silencing significantly affects proliferation and invasiveness of ERMP1-positive cancer cells, suggesting a role of the protein in growth and propagation of cancer cells. We measured such effect only *in vitro*, since we could not obtain stable ERMP1 knock down, likely due to low cell viability.

Second, ERMP1 expression is affected by menadione- and thapsigargin-induced stresses, and by hypoxia stimuli mimicking conditions typically encountered by cancer cells in their natural environment, which are acknowledged stimuli of UPR. Third, loss of ERMP1 expression during thapsigargin stress significantly decreases the level of PERK activation/phosphorylation and the expression of the stress marker GRP78/BiP, thereby suggesting an impairment of UPR activation. Upon stress sensing, tumor cells activate UPR and GRP78/BiP, which initiate the cyto-protective aspects and promote the ability of cancer cells to survive the hostile microenvironment. Fourth, ERMP1 silencing in cells exposed to 1% O_2_ hypoxia, a stimulus associated to mitochondrial delivery of oxidants and hence sustaining oxidative stress, leads to inhibition of the Nrf2-mediated anti-oxidant response. Under ER stress, the PERK/Nrf2 signaling pathway coordinates the interplay between UPR and oxidative stress caused by the accumulation of ROS. NrF2, a direct phosphorylation substrate of activated PERK, migrates to the nucleus to activate gene encoding anti-oxidant proteins and detoxifying enzymes, fighting perturbations in redox homeostasis [16, 17]. Fifth, ERMP1 silencing under hypoxia leads to reduction of accumulation of HIF-1, the master transcription factor instructing cells to respond to hypoxic stress. Hypoxia, a common feature of several solid advanced cancers, has been associated with both UPR and oxidative stress due to mitochondria deregulation [18, 19]. Such stimulus activates both UPR via the PERK downstream pathway and the HIF-s pathway [20]. These findings suggest that ERMP1 is involved in the ability of cancer cells to respond to hypoxia as a stress and oxidative signal, indicating that both HIF-1 and Nrf2 transcription factors are affected by ERMP1 RNA interference. In keeping with this idea, hypoxic and oxidative signals are often common in aggressive cancers, they trigger joint molecular pathways and converge on HIF-1 as a central player [21–23]. Indeed, HIF-1 transcription factor is a redox-sensitive protein, due to its regulation by a Fe-dependent prolyl hydroxylase that, upon ROS-mediated inactivation leads to HIF-1 stabilization. It is therefore likely that EMRP1, mediating UPR in cancer cells, acts upstream to both hypoxic and oxidative stress signals, thus behaving as a central molecular hub in stressful circumstances.

An unsolved question is why ERMP1 protein expression level decreases under ER stresses that activate UPR, whereas its transcriptional level remains unaltered. Although not investigated, this phenomenon might be explained by an altered turnover of the protein caused by PERK activation. Indeed, under ER stress, the eukaryotic initiation factor 2 alpha (eIF2α) is phosphorylated by PERK and it inhibits global protein synthesis while preserving a selective translation of a small number of mRNAs involved in UPR, including the activating transcription factor 4 (ATF4) [1, 5]. Under this condition, ERMP1 could be rapidly consumed in the attempt to re-establish ER normal state but its expression level is not restored, as a consequence of eIF2α –P-mediated attenuation of protein translation.

Overall, our study highlights ERMP1 as a novel target for anti-cancer therapies. Since the protein is localized intracellularly, it is amenable to the development of nanocarrier-based approaches able to drive ERMP1-specific silencing tools within the cells [24, 25]. Moreover, its involvement in UPR could foster the development of novel drugs targeting UPR. Anti-cancer approaches affecting the UPR pathway are having initial successes in clinical studies [1, 5]. The therapeutic potential of drugs targeting the UPR components in cancer mainly involves two approaches, both promoting cell deaths: induction of accumulation of misfolded protein in ER to overload the unfolded protein response so as to induce the pro-apoptotic pathways or inhibition of UPR adaptive response and pro-survival pathway. Our data provide the rationale for the design of novel ERMP1-targeting drugs that could act by inhibiting the UPR initial adaptive response of cancer cells and impair cell survival. Moreover, since high expression of GRP78/BiP protect cancer cells from the cytotoxic effects of several chemotherapeutic agents [26, 27], targeting ERMP1 could also weaken GRP78/BiP-dependent chemoresistance mechanisms of cancer cells.

A concern that may limit the exploitation of UPR in anti-cancer therapy is that most components of this pathway can be activated by a variety of physiological and pathological conditions, not necessarily related to cancer, as well as by different pharmacological agents [1]. Thus, the use of drugs affecting UPR may lead to unwanted toxic effects in non-cancerous cells suffering from various stresses. In this context ERMP1, being highly expressed in cancer cells might be used to overcome the current limit of UPR targeting drugs.

## MATERIALS AND METHODS

### Reagents, cell cultures and TMAs

Unless specified, all reagents were obtained from Sigma. His-tagged recombinant ERMP1 domains were generated in E. coli as described [10]. Human cells were obtained from the ATCC collection and, unless differently stated, cultured under ATCC recommended conditions. Anti-HIF-1 antibody was from BD Transduction Laboratories. Anti-Nrf2, anti-actin and anti-p-PERK antibodies were from Santa Cruz Biotechnology. Anti-PERK antibody was from Cell Signaling Technology. The four TMAs used were produced within previous Biobank activities at Pathology of the University Hospital of Basel. All activities were conducted previous acquirement of the local ethical committee permission. A commercial TMA (MNO961, Pantomics, Richmond, CA, USA) carrying normal samples from 33 organs was also used to assess ERMP1 staining.

### IHC analysis

For each cancer entity different histological stages and grades are represented except for NSCLC where all samples were diagnosed as pT1 or pT2, and pN0 (Supplementary Table S1). TMA production and IHC staining were performed essentially as previously described [11, 28, 29]. Briefly, formalin-fixed, paraffin-embedded tissue blocks of surgical resections were retrieved from the archives of the Institute of Pathology, University Hospital Basel and the Institute of Clinical Pathology Basel, Switzerland. Cancer and normal samples were arrayed in parallel on the same TMA slides and analyzed simultaneously. One tissue cylinder with a diameter of 0.6 mm was punched from morphologically representative tissue areas, mostly central tumor areas and rather away from the infiltrating tumor border. Clinico-pathologic data from the corresponding series of cancer cases were obtained from archived files. In breast cancer tissue, positivity to human epidermal growth factor receptor 2 (HER2), progesterone and estrogen receptor were annotated as previously described [30]. Briefly ER and PR staining was scored semiquantitatively using the proportion of positive tumor cells over total tumor cells (%-positivity, ranging from 0% to 100% at 5% intervals). Any intensity of staining of more than 10% was considered positive. FISH analysis was used to detect the amplification of HER2. A tumor was considered amplified if the ratio of oncogene / centromere was ≥ 2.0. In lung cancer, positivity to Ki67 was also scored semiquantitatively using the proportion of positive tumor cells over total tumor cells (%-positivity, ranging from 0% to 100% at 5% intervals). In CRC, Sanger sequencing analysis for BRAF and K-RAS gene alterations, was performed as previously described [31].

Concerning the scoring of ERMP1 staining slides were screened semiquantitavely for the percentage and the intensity of the signal for ERMP1. At least 100 cells were counted for each punch. Intensity of the signal was graded semiquantitavely in 4 groups from 0 (no positivity) to 3 (strong positivity). A case was considered low positive if showed a positive signal between 10% and 33% of cells, moderate positive between 33 and 66% and strong positive more than 66%. Negative control samples were prepared by using an irrelevant isotype control antibody and/or by omitting the primary antibody. The study was approved by the Human Research Ethic Committee of University Hospital of Basel (Ethikkommission Nord-und Zentralschweiz, reference number EK 322/13).

### Cell transfection

A pcDNA3.1D (Invitrogen) derivative plasmid encoding ERMP1 full length cDNA was generated and sequence verified. Transfection was assessed in HeLa cells using a protocol already described [10].

### Environmental stimuli

The influence of oxidative stress on ERMP1 expression was assessed in SK-BR-3 and MCF7 cells using DME medium without sodium bicarbonate supplemented with 25 mM HEPES, 10 mM sodium bicarbonate supplemented with 3μM menadione, or 0.5 μM thapsigargin, or brought to pH 6.8 (acidic pH stress). Moreover the influence of CAFs conditioned medium was tested. To obtain CAFs conditioned medium, human mammary fibroblasts were grown to sub-confluence, treated for 24 hours with 10 ng/ml transforming growth factor β (TGF-β) (Peprotech) and then starved for additional 24 hours before collecting the conditioned medium.

### ERMP1 silencing

*ERMP1* was silenced in the indicated cancer cell lines with suboptimal concentrations (1 nM) of 4 commercially available *ERMP1*-specific siRNAs (#SI04291497, #SI03226006, #SI03132311, #SI00460873, QIAGEN) or irrelevant siRNA (AllStars Negative Control siRNA, QIAGEN) using the HiPerfect transfection reagent (QIAGEN) following the manufacturer's protocol. Loss of *ERMP1* expression was verified by q-RT-PCR and WB (after 48 or 72 hours).

### Electrophoresis and western blot analysis

Cells were suspended in Laemmli electrophoresis buffer (without β-mercaptoethanol and bromophenol blue) and assayed for protein content by the bicinchoninic acid (BCA) and the Bradford method. After that each sample were additioned with β-mercaptoethanol and separated by SDS-PAGE. Subsequently gels were electroblotted onto Polyvinylidene difluoride (PVDF) membranes (Millipore) and the blots were incubated with anti-ERMP1, anti-Nrf2, anti-Actin, anti-HIF-1, anti-PERK and anti-p-PERK. After incubation with secondary antibodies, the blotting was developed by using the ECL plus immunodetection system (Bio-Rad) and the chemiluminescence was visualized by UVP (Ultra-Violet Products) Ltd Chemidoc-it 500 Imaging System. Quantitative analysis of the spots was carried out by Kodak MI software.

### RNA extraction and q-RT-PCR analysis

RNA extraction from clinical samples and cell lines was performed using the RNeasy mini kit (QIAGEN) and 500 ng of it were reverse transcribed using Superscript III Reverse Transcriptase (Life Technologies) with oligo dT. Triplicate cDNA samples from each cell line (equal to 50 ng RNA/sample) were subjected to real-time q-PCR to assess the relative ERMP1 (Quantitect^®^ Primer Assay for Human ERMP1, QIAGEN) and GRP78/BiP (primers: 5′-CGTGGATGACCCGTCTGTG-3′ and 5′-CGTCTTT GGTTGCTTGGC-3′) transcript levels using the Quantitect^®^ SYBR Green PCR kit (QIAGEN). MAPK, actin (Quantitect^®^ Primer Assay for Human actin or MAPK, QIAGEN), or β2-microglobulin (primers: 5′-AGTATGCCTGCC GTGTGAAC-3′ and 5′-GCGGCATCTTCAAACCTC CA-3′) were used as an internal normalization controls, respectively. Data were analyzed with the One-Step Plus q-RT-PCR equipment (Applied Biosystems).

### Measurement of intracellular ROS

Cells were cultured for 24 hours in starvation medium at 20% O_2_ and at 1% O_2_, after that cells were treated with 5 μg/mL of 2′,7′–dichlorofluorescin diacetate (DCFDA) for 15 minutes, washed with PBS and lysed in RIPA buffer. The fluorescence was immediately detected by spectrophotometric analysis at 510 nm.

### Confocal microscopy

Cells were plated on microscope coverslips, stained with the anti-ERMP1 antibodies at the appropriate concentrations, using a previously described protocol [11]. For detection, Alexafluor 488-labeled goat anti-mouse antibodies and DAPI were used.

### Phenotypic assays

Cell viability and apoptosis were measured by the MTT (Promega) and the Annexin V-PI assays (Sigma). Assays were performed in 96w-well plates according to the manufacturer's instruction. Cell invasiveness was assessed by the Boyden chamber assay. The indicated cell lines (2000/well) were placed into 24-well plates containing 500 μl of DMEM complete medium. After ON incubation at 37°C, non-invading cells were removed mechanically using cotton swabs, and micro-porous membrane containing the invaded cells was fixed in 96% methanol and stained with Diff-Quick staining solutions. Invasiveness was evaluated by counting the cells that migrated towards the lower surface of the filters (10 randomly chosen fields for each filter). Each experiments was carried out in triplicate and averaged from at least 3 independent experiments.

### Statistical analysis

Statistical analysis of the phenotypic data was performed using two-tailed Student's *t*-test. The statistical association between the clinico-pathological variables and ERMP1 IHC staining was assessed with the chi-square test. *P*-values ≤ 0.05 were considered significant.

## SUPPLEMENTARY MATERIALS FIGURES AND TABLES




