# Supplementary material for: ERMP1, a novel potential oncogene involved in UPR and oxidative stress defense, is highly expressed in human cancer
Source: Oncotarget. 2016 Aug 23;7(39):63596–610. doi: 10.18632/oncotarget.11550 (PMC5325388; doi:10.18632/oncotarget.11550)
Supplement: Supplementary file 1 [file oncotarget-07-63596-s001.pdf]

ERMP1, a novel potential oncogene involved in UPR and oxidative stress defense, is highly expressed in human cancer

Supplementary Materials

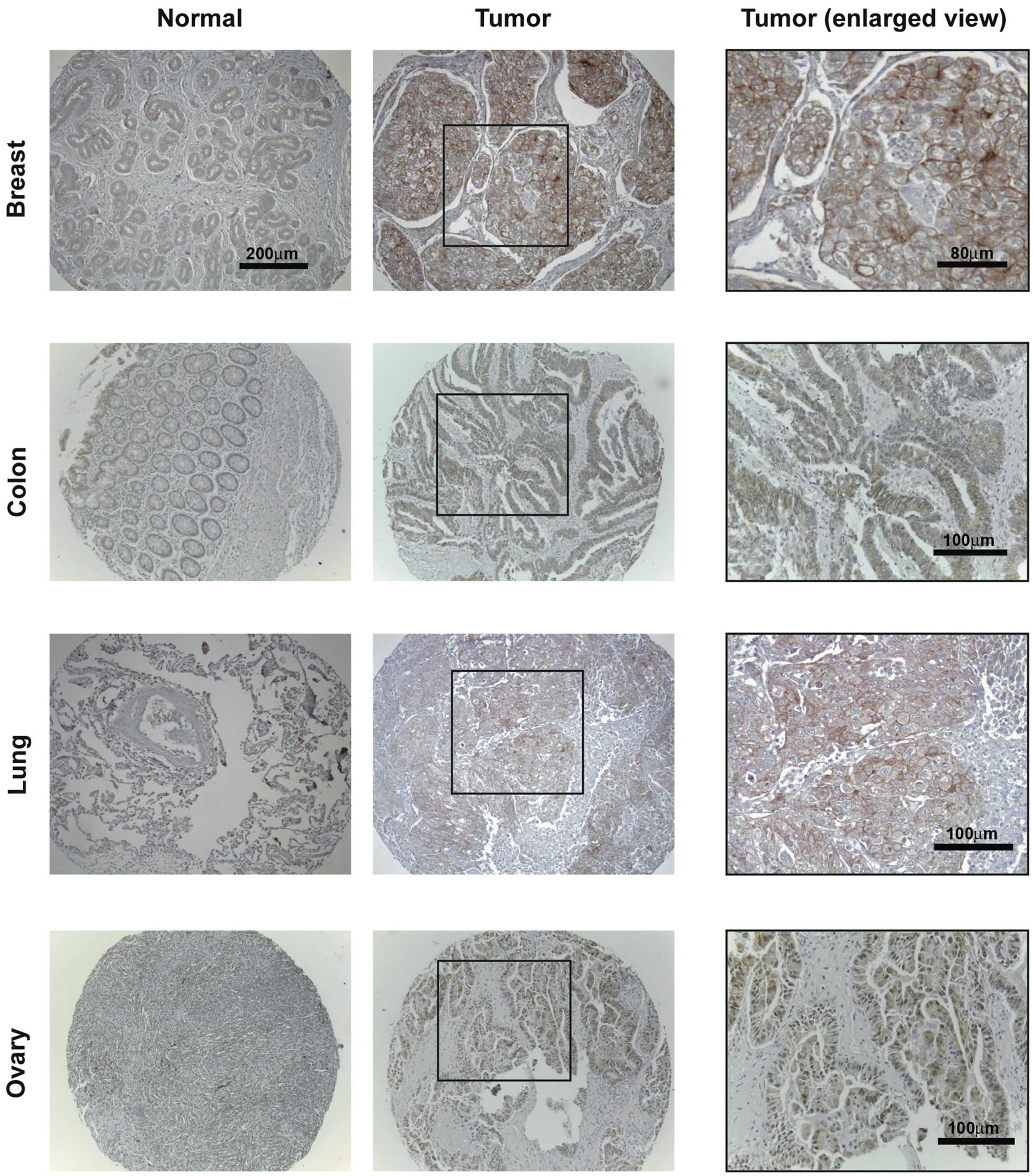

Supplementary Figure S1: The anti-ERMP1 polyclonal antibody specifically detects its target protein in cancer samples. Immunostaining of cancerous and normal samples from breast, colon, lung and ovary with the pAb687-YOM polyclonal antibody.

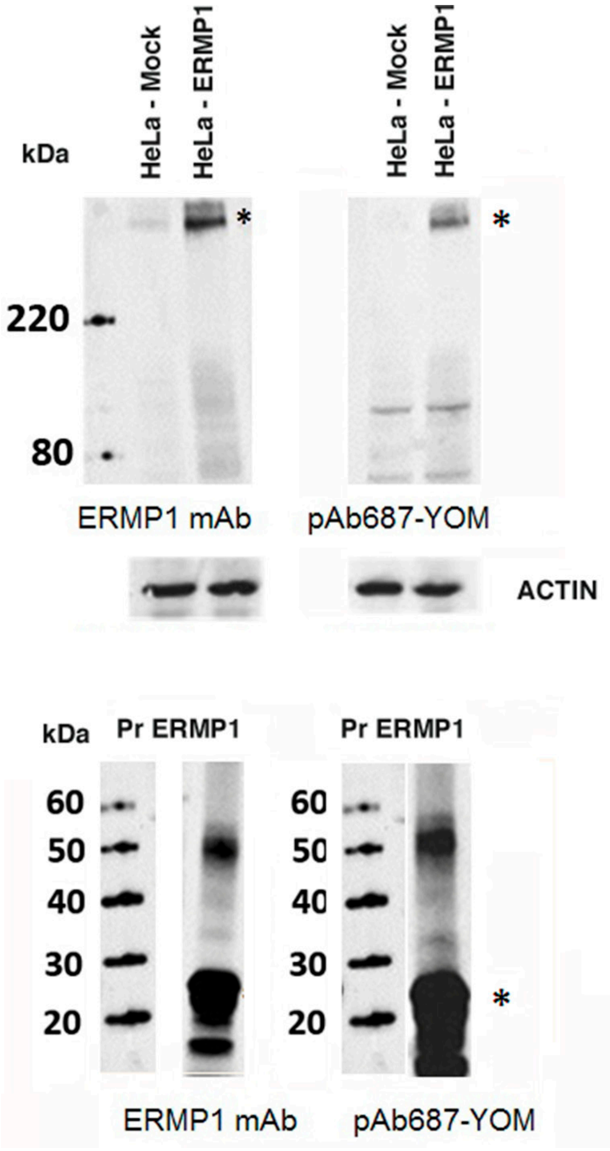

Supplementary Figure S2: Western blot analysis of ERMP1 transfected Hela cells and ERMP1 recombinant protein using the anti-ERMP1 pAb and mAb. (**A**) HeLa cells were transfected with ERMP1 coding plasmid or the empty plasmid and, 48 h later total extracts were obtained,separated by SDS-PAGE (25 µg/lane, corresponding to approximately 0.5×10^6 cells) and subjected to Western blot with the polyclonal and monoclonal antibodies against ERMP1. An anti-actin antibody was used as internal loading control. (**B**) The recombinant ERMP1 domain (amino acid 1–204) was separated by SDS-PAGE (50 ng/lane). Molecular weight standards are on the left.

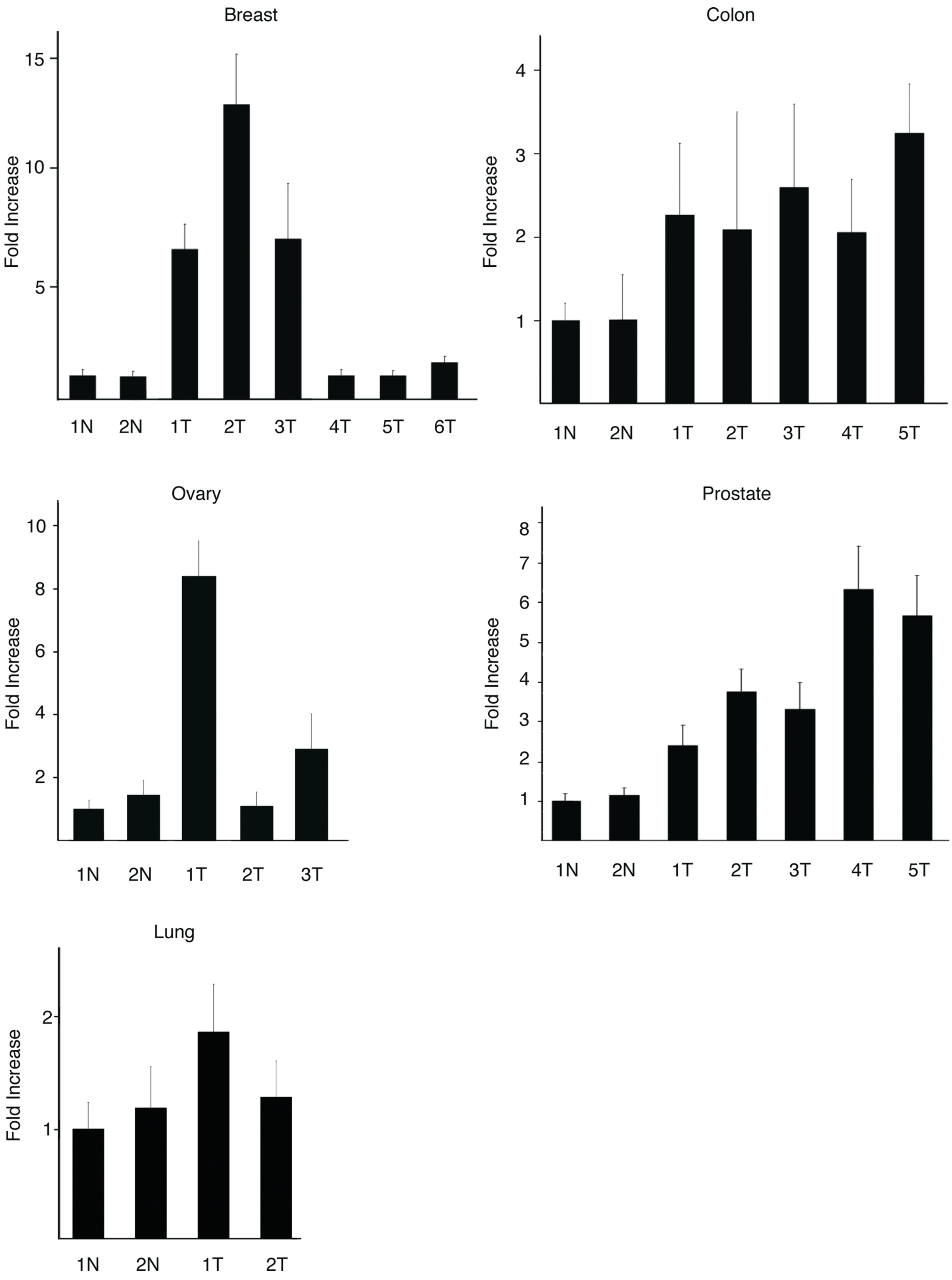

Supplementary Figure S3: ERMP1 expression analysis in clinical samples by q-RT-PCR. Total RNA was purified from tumor (T) and normal (N) clinical samples and the ERMP1 transcript level was quantified by q-RT-PCR vs MAPK or ACTIN housekeeping genes and further normalized to the ERMP1 level in normal samples (the sample having lower transcript level).

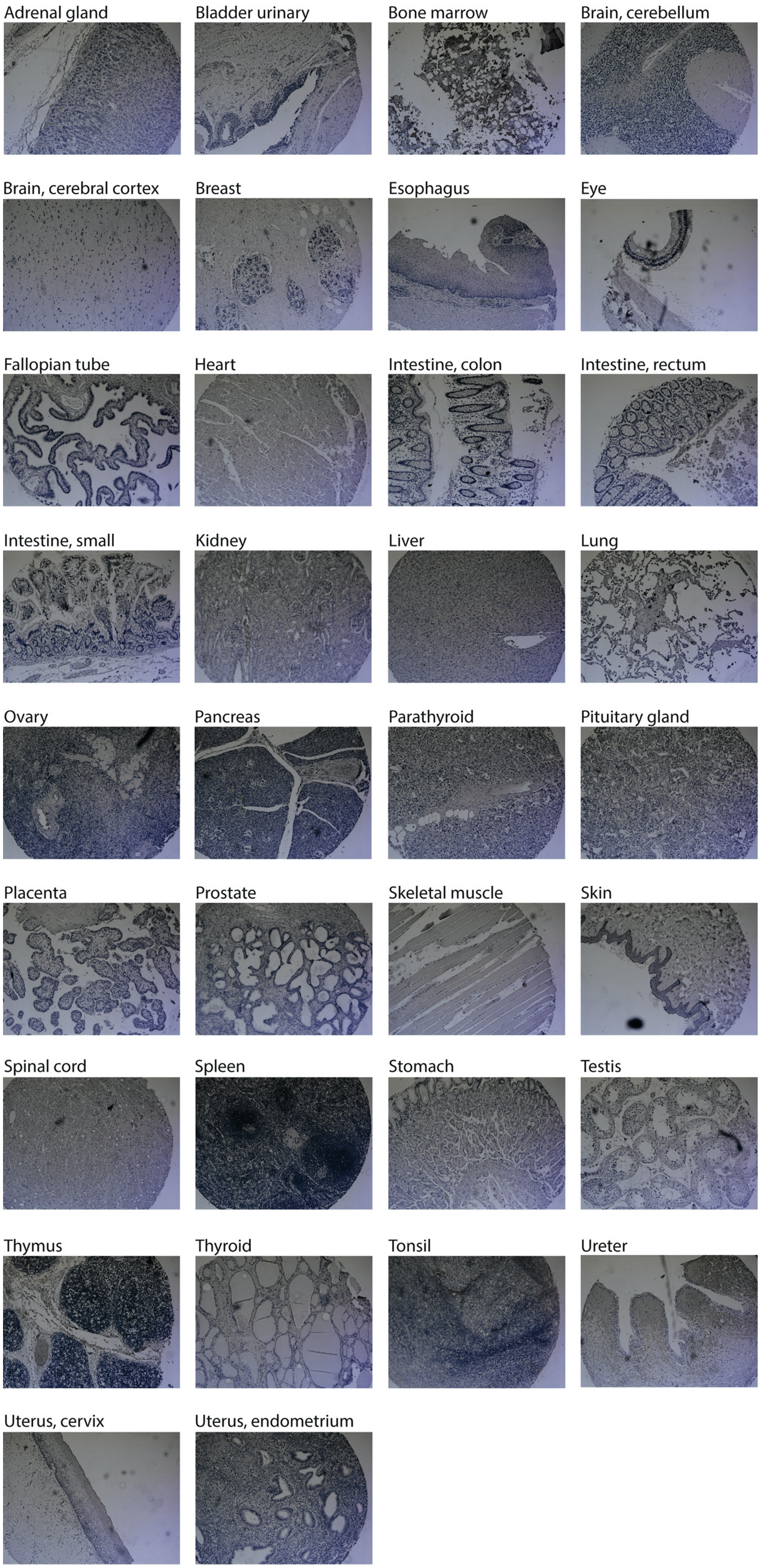

Supplementary Figure S4: ERMP1 expression in healthy human tissues. IHC immunostaining images of 33 normal human tissues detected by anti-ERMP1 mAb by IHC. Representative images of each tested tissue are reported.

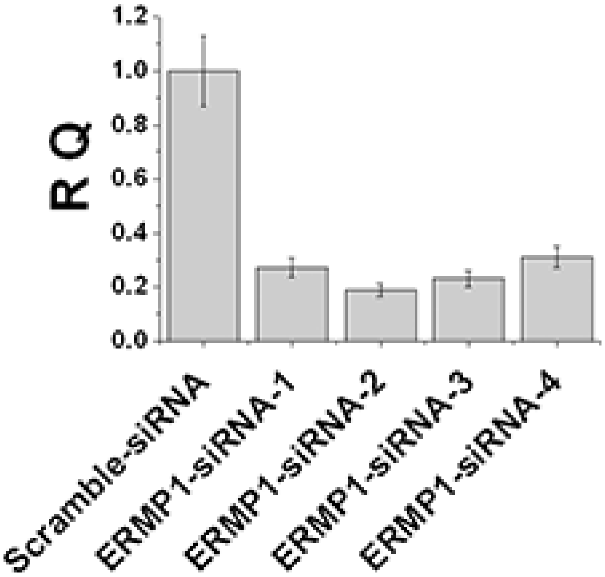

Supplementary Figure S5: q-RT-PCR analysis of ERMP1 knockdown. SK-BR-3 cells were transfected with four ERMP1-specific siRNAs or and irrelevant siRNA control (1 nM) and 72 hours later total RNA isolated, reverse transcribed and subjected to real time PCR. All tested siRNAs significantly inhibit ERMP1 transcription.

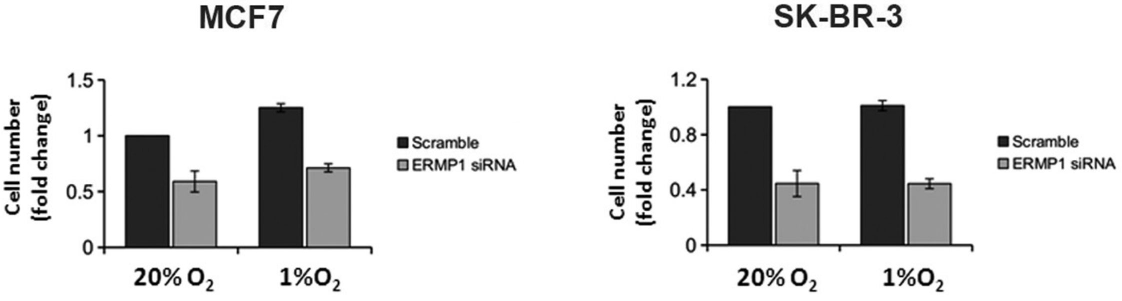

Supplementary Figure S6: ERMP1 silencing affects cell proliferation under hypoxic and normoxic conditions. MCF7 and SK-BR-3 cells were transfected with ERMP1-specific siRNAs or scrambled siRNA. After 48 hours cells were plated in 96W cell plates (2×10^4 cells per well) and incubated for 24 h at 20% O_2 or 1% O_2. Proliferation was assessed by the MTT assay.

Supplementary Table S1: Design of the ovary cancer TMA. See Supplementary_Table_S1.
